# Prediction of Phenotypic Antimicrobial Resistance Profiles From Whole Genome Sequences of Non-typhoidal *Salmonella enterica*

**DOI:** 10.3389/fmicb.2018.00592

**Published:** 2018-03-27

**Authors:** Saskia Neuert, Satheesh Nair, Martin R. Day, Michel Doumith, Philip M. Ashton, Kate C. Mellor, Claire Jenkins, Katie L. Hopkins, Neil Woodford, Elizabeth de Pinna, Gauri Godbole, Timothy J. Dallman

**Affiliations:** ^1^National Institute for Health Research Health Protection Research Unit in Gastrointestinal Infections, University of Liverpool, Liverpool, United Kingdom; ^2^Bacteriology Reference Department, National Infection Service, Public Health England, London, United Kindom; ^3^Department of Pathobiology and Population Sciences, Royal Veterinary College, London, United Kingdom; ^4^London School of Hygiene & Tropical Medicine, London, United Kingdom

**Keywords:** antimicrobial resistance, multidrug resistance, non-typhoidal *Salmonella enterica*, whole genome sequencing, public health surveillance, One Health

## Abstract

Surveillance of antimicrobial resistance (AMR) in non-typhoidal *Salmonella enterica* (NTS), is essential for monitoring transmission of resistance from the food chain to humans, and for establishing effective treatment protocols. We evaluated the prediction of phenotypic resistance in NTS from genotypic profiles derived from whole genome sequencing (WGS). Genes and chromosomal mutations responsible for phenotypic resistance were sought in WGS data from 3,491 NTS isolates received by Public Health England’s Gastrointestinal Bacteria Reference Unit between April 2014 and March 2015. Inferred genotypic AMR profiles were compared with phenotypic susceptibilities determined for fifteen antimicrobials using EUCAST guidelines. Discrepancies between phenotypic and genotypic profiles for one or more antimicrobials were detected for 76 isolates (2.18%) although only 88/52,365 (0.17%) isolate/antimicrobial combinations were discordant. Of the discrepant results, the largest number were associated with streptomycin (67.05%, *n* = 59). Pan-susceptibility was observed in 2,190 isolates (62.73%). Overall, resistance to tetracyclines was most common (26.27% of isolates, *n* = 917) followed by sulphonamides (23.72%, *n* = 828) and ampicillin (21.43%, *n* = 748). Multidrug resistance (MDR), i.e., resistance to three or more antimicrobial classes, was detected in 848 isolates (24.29%) with resistance to ampicillin, streptomycin, sulphonamides and tetracyclines being the most common MDR profile (*n* = 231; 27.24%). For isolates with this profile, all but one were *S*. Typhimurium and 94.81% (*n* = 219) had the resistance determinants *bla*_TEM-1,_ *strA-strB, sul2* and *tet*(A). Extended-spectrum β-lactamase genes were identified in 41 isolates (1.17%) and multiple mutations in chromosomal genes associated with ciprofloxacin resistance in 82 isolates (2.35%). This study showed that WGS is suitable as a rapid means of determining AMR patterns of NTS for public health surveillance.

## Introduction

*Salmonella enterica* subspecies *enterica* is responsible for 99% of salmonellosis cases in humans and animals, and can be further subdivided into the host-restricted typhoidal salmonellae and the more generalist non-typhoidal salmonellae (NTS) (Langridge et al., 2015; Wain et al., 2015). As host-adapted or generalist organisms, NTS can be transferred from animals to humans causing zoonotic infections and therefore fall under the World Health Organization’s One Health approach. Globally, NTS were estimated to cause 93.8 million enteric infections resulting in 155,000 deaths annually (Majowicz et al., 2010), and in the United Kingdom they are the third most common cause of bacterial gastroenteritis (Tam et al., 2012). Although NTS symptoms are often limited to the gastrointestinal tract, invasive disease can occur, especially in high-risk groups such as immunocompromised patients and the elderly (Parry et al., 2013). Invasive disease has also been observed in several low-income settings (Kingsley et al., 2009; Feasey et al., 2016; Ashton et al., 2017) and was estimated to result in 3.4 million cases and 681,000 deaths worldwide in 2010, with the heaviest burden on the African continent (Ao et al., 2015).

While the use of antimicrobial agents to treat invasive and severe gastrointestinal cases has decreased mortality rates for NTS infections, and veterinary antimicrobial therapy has lowered the risk of zoonoses, these interventions have come with a price. Increased use of the traditional first-line drugs ampicillin, chloramphenicol, streptomycin, sulphonamides and tetracycline quickly led to the emergence of ACSSuT-type *S*. *enterica* serovar Typhimurium strains in the 1980s, resistant to exactly these drugs (Threlfall et al., 1996; Boyd et al., 2002). Resistance to fluoroquinolones, introduced to circumvent this problem, developed as a consequence of the veterinary use of enrofloxacin (Threlfall et al., 1997). NTS strains resistant to extended-spectrum cephalosporins, an alternative to fluoroquinolones for the treatment of invasive disease, have been detected throughout Europe since the 1990s (Tassios et al., 1999; Villa et al., 2002; Burke et al., 2014). By 2015, 29.3% of the NTS isolates in the European Union were categorized as multidrug-resistant (MDR) (EFSA, 2017). More recently, the spread of an extensively drug-resistant strain of *S*. Kentucky, non-susceptible to ciprofloxacin, extended-spectrum cephalosporins, carbapenems, most aminoglycosides, trimethoprim-sulfamethoxazole, and azithromycin, has sparked concern (Le Hello et al., 2013). Resistance to azithromycin has been reported in other NTS serovars (Villa et al., 2015; Nair et al., 2016). Acquired resistance to colistin, considered the antimicrobial of last resort for the treatment of many MDR Gram-negative pathogens, has also been detected in NTS (Doumith et al., 2016).

Due to the association of MDR NTS infection with increased mortality and higher costs to the healthcare system, determination of antimicrobial resistance (AMR) profiles is an essential part of NTS surveillance in reference laboratories. Phenotypic serotyping and phage typing at Public Health England’s (PHE) Gastrointestinal Bacteria Reference Unit (GBRU) has been replaced by the routine implementation of whole genome sequencing (WGS) for identification and surveillance of *Salmonella* since April 2014 (Ashton et al., 2016). As well as providing information about phylogenetic relationships between isolates, the sequencing data can be used to identify resistance determinants and therefore constitutes a rapid alternative to monitor emerging trends in AMR patterns of NTS. With this study, we sought to evaluate the suitability of inferring AMR profiles from genotype in NTS in comparison with traditional phenotypic susceptibility testing.

## Materials and Methods

### Bacterial Isolates

Between April 2014 and March 2015, PHE received 7,009 NTS *S*. *enterica* subspecies *enterica* isolates for surveillance purposes. After deduplication of outbreak cases and exclusion of isolates with WGS of insufficient quality, results of phenotypic susceptibility testing and genotypic profiling were available for 3,491 isolates (49.81%). These comprised 227 different serovars plus 66 isolates that could not successfully be subtyped to serovar level. GBRU’s routine phenotypic testing strategy for surveillance of NTS attempts to maximize the detection of AMR by focussing on serovars known to have high resistance rates. This leads to an under-representation of some serovars, such as *S*. Enteritidis, and an over-representation of others, such as *S*. Infantis and *S*. Kentucky, in this dataset. Amongst the isolates included in the analysis, the ten most common serovars were *S*. Typhimurium (23.69%, *n* = 827), *S*. Enteritidis (8.42%, *n* = 294), *S*. Virchow (4.01%, *n* = 140), *S*. Stanley (3.98%, *n* = 139), *S*. Newport (3.75%, *n* = 131), *S*. Infantis (3.47%, *n* = 121), *S*. Kentucky (3.12%, *n* = 109), *S*. Oranienburg (2.06%, *n* = 72), *S*. Java (2.03%, *n* = 71) and *S*. Saint-Paul (1.78%, *n* = 62). The majority (*n* = 3487) of isolates were of human origin, three were derived from food and one from an unknown source.

### Whole Genome Sequencing

Sequencing libraries were prepared from extracted genomic DNA using the Nextera XT DNA Sample Preparation kit (Illumina, Cambridge, United Kingdom). Short-read sequence fragments of 100 bp were produced by paired-end sequencing on an Illumina HiSeq platform (Illumina, Cambridge, United Kingdom). FASTQ sequences were deposited in the NCBI Short Read Archive under the BioProject PRJNA315192. Short read archive accession numbers are available in **Supplementary Table S1**.

### Serovar Prediction

Serovars were inferred from the sequencing data using the seven-gene MLST and eBurst Group approach (Achtman et al., 2012; Ashton et al., 2016). Traditional serotyping was not performed.

### Detection of Antimicrobial Resistance Determinants

For the identification of AMR determinants, the ‘Genefinder’ algorithm was employed, which maps the sequencing reads to a set of reference sequences using Bowtie 2 followed by generation of an mpileup file using Samtools (Langmead and Salzberg, 2012). To establish the presence of the reference sequence or nucleotide variations within the read set, a positive match had to meet the following criteria: query coverage 100%, base-call variation > 85% and nucleotide identity > 90%.

The reference database used included acquired genes and mutations known to confer resistance to β-lactams (including penicillins, 2nd-, 3rd- and 4th-generation cephalosporins and carbapenems), fluoroquinolones, aminoglycosides, sulphonamides, tetracyclines, trimethoprim and phenicols (Day et al., 2017b; Sadouki et al., 2017). Variants of β-lactamase genes were identified with 100% identity based on reference sequences downloaded from the Lahey^1^ or NCBI β-lactamase data resources^2^. Further reference sequences for acquired resistance genes were obtained from the Comprehensive Antimicrobial Resistance Database^3^ and the Resfinder datasets^4^. Chromosomal mutations were limited to previously published variations within the quinolone resistance-determining regions (QRDRs) of *gyrA* and *parC*.

### Antimicrobial Susceptibility Testing

Isolates were recovered from the PHE archive and retrospective susceptibility testing was performed and interpreted using EUCAST breakpoints and screening concentrations^5^. For the purpose of epidemiologically screening the large numbers of *S. enterica* isolates received by the reference laboratory, agar dilution with Mueller–Hinton agar was used to determine breakpoint values of ampicillin, cefotaxime, ceftazidime, cefpirome, ertapenem, chloramphenicol, gentamicin, streptomycin, tobramycin, sulphonamides, tetracycline, trimethoprim and ciprofloxacin. Decreased susceptibility (MIC 0.06–0.25 mg/L) and resistance (MIC > 0.5 mg/L) were distinguished for ciprofloxacin. If required, MICs were confirmed by Etest^®^ (bioMérieux, Marcy-l’Étoile, France) or by agar dilution. To aid detection of OXA-48-like carbapenemases and acquired AmpC genes, breakpoint testing of temocillin and cefoxitin, respectively, was included in the panel.

### Statistical Analysis

Comparisons were made between the prevalence of resistance determinants associated with isolates, for which a travel history was available, and those for which there was no information about recent travel. Travel destinations were grouped according to the United Nations geoscheme. Statistical significance was assessed using the chi-square test. A *p*-value ≤ 0.05 was considered statistically significant. Statistical analysis was performed using R’s chisq.test function.

## Results

### Comparison Between Phenotypic and Genotypic AMR Profiles

Phenotypic and genotypic AMR profiling was highly correlated, with the profiles of 3,415 isolates (97.82%) being entirely in agreement for both approaches for all 15 antimicrobials from nine different classes. For the 76 isolates with discordant results, the genotype wrongly predicted pan-susceptibility for one isolate (1.32%). This isolate was phenotypically resistant to one antimicrobial. For a further 64 discrepant isolates (84.21%), the mismatch was based on false or missing prediction of resistance to a single antimicrobial, and for 11 (14.47%) based on two antimicrobials.

Overall, 88 (0.17%) out of a possible 52,365 isolate/antimicrobial combinations did not match (**Table 1**). Of these discrepant results, 69/88 (78.41%) constituted major errors (MEs), i.e., isolates were genotypically predicted to be resistant but showed phenotypic susceptibility, rather than very major errors (VMEs), which were genotypically susceptible but phenotypically resistant. The largest fraction of the 88 mismatches could be attributed to streptomycin (*n* = 59, 67.05%), 51 of these were MEs. Sensitivity of resistance prediction from genotype was ≥95% for all antimicrobials except temocillin. However, only a single isolate was found to be phenotypically resistant to temocillin. Specificity of prediction exceeded 98% for all fifteen antimicrobials tested.

**Table 1:** Comparison of phenotypic antimicrobial susceptibility testing and genome-derived resistance prediction for non-typhoidal *Salmonella enterica* (*n* = 3491).

Antimicrobial	Phenotype: susceptible		Phenotype: resistant		Sensitivity (%)	Specificity (%)
	Genotype: resistant	Genotype: susceptible	Genotype: resistant	Genotype: susceptible		
Ampicillin	1	2742	747	1	99.87	99.96
Temocillin	0	3490	0	1	0	100
Cefoxitin	0	3471	19	1	95.0	100
Cefotaxime	0	3434	57	0	100	100
Ceftazidime	0	3444	47	0	100	100
Cefpirome	0	3444	47	0	100	100
Ertapenem	0	3481	10	0	100	100
Chloramphenicol	4	3284	201	2	99.01	99.88
Gentamicin	1	3351	138	1	99.28	99.97
Streptomycin	51	2821	613	8	98.71	98.22
Tobramycin	2	3392	97	0	100	99.94
Sulphonamides	2	2661	828	0	100	99.92
Tetracycline	6	2568	917	0	100	99.77
Trimethoprim	1	3185	301	4	98.69	99.97
Ciprofloxacin	1	3352	137	1	99.28	99.97

*Values shown designate the number of isolates. For ciprofloxacin only isolates with a MIC > 0.5 mg/L are shown.*

### Resistance to β-lactams

Of the 3,491 isolates in this study, 749 (21.46%) carried genes conferring resistance to β-lactam antibiotics (**Supplementary Table S2**). The most common genes were the penicillinase-encoding *bla*_TEM-1_ (*n* = 603) and *bla*_PSE-1_/*bla*_CARB-2_ (*n* = 75). Additionally, other TEM-type β-lactamase genes were detected in 36 isolates, including *bla*_TEM-117_ (*n* = 12) and *bla*_TEM-135_ (*n* = 7). The single ME associated with predicted ampicillin resistance was due to the presence of *bla*_TEM-1_ without phenotypic consequences. Seven isolates (0.20%) carried OXA-type class D β-lactamases. Of these, four were found in *S*. Typhimurium and two in *S*. Kentucky.

Genes for CTX-M-type extended-spectrum β-lactamases (ESBLs) were present in 41 isolates (1.17%), most commonly *bla*_CTX-M-9_ (*n* = 10) and *bla*_CTX-M-55_ (*n* = 9). Twenty of these were *S*. Typhimurium and five *S*. Kentucky. Additionally, four isolates carried the *bla*_SHV -12_ ESBL gene. No ESBL genes were detected in *S*. Enteritidis. Combinations of penicillinase and ESBL genes occurred in 16 isolates, most frequently *bla*_TEM-1_ with *bla*_CTX-M-55_ (*n* = 9).

Sixteen isolates (0.46%), seven of these *S*. Typhimurium and one *S*. Kentucky, had the acquired AmpC resistance gene *bla*_CMY -2_. Carbapenemase genes were not detected.

### Resistance to Quinolones

Multiple mutations in the QRDR of the DNA gyrase subunit gene *gyrA* in combination with multiple mutations in the DNA topoisomerase gene *parC* are expected to confer full ciprofloxacin resistance (MIC > 0.5 mg/L) and were observed in 82 isolates (2.35%) (**Table 2**). The most common combinations were either *gyrA*[83:S-F;87:D-Y] (*n* = 41) or *gyrA*[83:S-F;87:D-N] (*n* = 25) in conjunction with *parC*[57:T-S;80:S-I]. For *S*. Kentucky, multiple QRDR mutations were identified in 77 isolates. Neither *S*. Typhimurium nor *S*. Enteritidis had any of these mutations (**Supplementary Table S2**).

**Table 2:** Relationship between decreased ciprofloxacin susceptibility (<CIP, MIC 0.06–0.25 mg/L), full ciprofloxacin resistance (>CIP, MIC > 0.5 mg/L) and the most common genotypic quinolone resistance determinants in non-typhoidal *Salmonella enterica*.

Number of isolates	Phenotype				Genotype
	<CIP		>CIP		
	S	R	S	R	
116	6	110	114	2	*gyrA*[87:D-Y]
51	1	50	42	9	*gyrA*[83:S-Y];*parC*[57:T-S]
51	4	47	51	0	*gyrA*[87:D-N]
47	0	47	43	4	*gyrA*[83:S-Y]
44	0	44	39	5	*parC*[57:T-S];*qnrS1*
41	0	41	0	41	*gyrA*[83:S-F;87:D-Y];*parC*[57:T-S;80:S-I]
37	1	36	28	9	*qnrS1*
34	2	32	34	0	*gyrA*[87:D-Y];*parC*[57:T-S]
32	0	32	31	1	*parC*[57:T-S];*qnrB19*
29	1	28	28	1	*gyrA*[83:S-F];*parC*[57:T-S]
25	0	25	1	24	*gyrA*[83:S-F;87:D-N];*parC*[57:T-S;80:S-I]
23	0	23	20	3	*gyrA*[83:S-F]
19	2	17	19	0	*gyrA*[87:D-G]
17	2	15	17	0	*gyrA*[87:D-G];*parC*[57:T-S]
12	6	6	12	0	*parC*[57:T-S];*qnrD*
12	0	12	9	3	*qnrB19*
11	0	11	0	11	*gyrA*[83:S-F;87:D-G];*parC*[57:T-S;80:S-I]
11	0	11	11	0	*gyrA*[87:D-N];*parC*[57:T-S]
8	0	8	8	0	*qnrA1*
7	1	6	3	4	*aac(6′)-Ib-cr*
4	0	4	0	4	*gyrA*[83:S-Y];*parC*[57:T-S];*qnrS1*
4	0	4	0	4	*gyrA*[87:D-Y];*qnrS1*
4	0	4	4	0	*parC*[57:T-S];*qnrB9*
4	0	4	4	0	*parC*[57:T-S];*qnrS2*
4	0	4	2	2	*qnrB6*
3	0	3	0	3	*gyrA*[83:S-F;87:D-G];*parC*[57:T-S;80:S-R]
3	0	3	1	2	*gyrA*[83:S-Y];*parC*[57:T-S];*qnrD*
3	0	3	0	3	*gyrA*[83:S-Y];*qnrS1*
2	0	2	2	0	*parC*[57:T-S];*qnrA1*
2	0	2	2	0	*parC*[57:T-S];*qnrB1*

*Values shown are the number of isolates. S, susceptible; R, Resistant.*

A further 599 isolates (17.16%) harbored determinants responsible for reduced susceptibility to ciprofloxacin (MIC 0.06–0.25 mg/L) with or without *parC* mutations. These included a single *gyrA* mutation in the QRDR (*n* = 430), most commonly *gyrA*[87:D-Y] (*n* = 155) or *gyrA*[83:S-Y] (*n* = 112) and/or one or multiple plasmid-mediated quinolone resistance (PMQR) genes (*n* = 195). The most frequent PMQR genes detected were *qnrS1* (*n* = 95) and *qnrB19* (*n* = 49). PMQR genes were rare in *S*. Kentucky (*n* = 1 compared with *n* = 188 for chromosomal mutations). One or more PMQR determinants in combination with a single *gyrA* mutation were found in twenty isolates. Of the isolates carrying both multiple *parC* and *gyrA* mutations, only one *S*. Indiana had additional PMQR genes, namely the efflux pump-encoding *oqxA* and *oqxB*.

Seven isolates carried the fluoroquinolone- and aminoglycoside-modifying *N*-acetyltransferase gene variant *aac(6′)-Ib-cr*, six of these in combination with other quinolone resistance determinants. Of the 138 isolates showing full ciprofloxacin resistance, nineteen carried a single *gyrA* mutation only, 17 a single *gyrA* mutation together with a PMQR gene, 20 had one or more PMQR genes and a single isolate carried *parC*[57:T-S] only (**Table 2**). The single ME associated with predicted ciprofloxacin resistance was based on the presence of *gyrA*[83:S-F;87:D-N] and *parC*[57:T-S;80:S-I] resulting in reduced susceptibility instead of full resistance.

### Resistance to Aminoglycosides

Genes predicted to confer resistance to streptomycin were detected in 728 isolates (20.85%): 436 had *strA-strB* only and 292 carried genes encoding aminoglycoside adenylyltransferases, most commonly *aadA2* (*n* = 189) and *aadA17* (*n* = 107) (**Supplementary Table S2**). Both *strA-strB* and an *aadA* variant were observed in 101 isolates. Of the 51 MEs associated with streptomycin resistance, 27 were due to the presence of *strA-strB* and twelve had *aadA2* and *aadA17* without phenotypic consequences.

All but eight of the total 3,491 isolates carried an aminoglycoside acetyltransferase *aac(6′)*-type gene. However, the majority either had the *aac(6′)-Iy* (*n* = 1997), more common in *S*. Enteritidis (*n* = 297), or *aac(6′)-Iaa* variant (*n* = 1486), more common in *S*. Typhimurium (*n* = 869) and *S*. Kentucky (*n* = 81). Of the 2,726 isolates carrying either of these two genes as the only aminoglycoside resistance determinant, only eleven showed phenotypic resistance to an aminoglycoside antimicrobial.

Aminoglycoside acetyltransferase *aac(3)* variants associated with resistance to gentamicin and tobramycin were detected in 130 isolates (3.72%), most notably *aac(3)-Id* (*n* = 50) and *aac(3)-IIa* (*n* = 36). *aac(3)-IVa*, which confers resistance to the veterinary aminoglycoside apramycin, was present in 24 isolates. No *aac(3)* variants were found in *S*. Enteritidis. Furthermore, the aminoglycoside adenylyltransferase gene *ant(2″)-Ia* (*n* = 12) and the aminoglycoside phosphotransferase genes *aph(4)-Ia* (*n* = 23) and *aph(3′)-IIa* (*n* = 10) were identified. None of these were present in *S*. Enteritidis or *S*. Kentucky. No 16S rRNA methyltransferase genes were detected. In the single isolate predicted to be resistant to gentamicin but showing phenotypic susceptibility, *aac(3)-IId* was observed. For prediction of tobramycin resistance, one ME was associated with the presence of *ant(2″)-Ia* and the second one with *aac(3)-IIa*.

### Resistance to Sulphonamides, Tetracyclines and Trimethoprim

Sulphonamide resistance genes were found in 830 isolates (23.78%): 490 carried *sul2*, 350 *sul1* and 75 *sul3* (**Supplementary Table S2**). Seventy-seven isolates had a combination of two different *sul* genes, most notably *sul1* and *sul2* (*n* = 37), and four isolates carried all three variants. Of the two MEs that occurred for the prediction of sulphonamide resistance, one was based on the presence of *sul2* and one on the presence of *sul1* without phenotypic consequences.

Tetracycline resistance genes occurred in 927 isolates (26.55%), mostly *tet*(A) (*n* = 843). Additional, less frequently encountered genes were the efflux pump-encoding *tet*(G) (*n* = 68), *tet*(C) (*n* = 10) and *tet*(D) (*n* = 5), and the ribosomal protection protein-producing *tet*(M) (*n* = 57). Fifty-six isolates carried a combination of two different genes, mainly *tet*(A) and *tet*(M) (*n* = 51). Five of the six isolates with predicted but not phenotypic tetracycline resistance harbored *tet*(M).

Trimethoprim resistance-conferring *dfrA* gene variants were identified in 302 isolates (8.65%), most commonly *dfrA12* (*n* = 84), *dfrA1* (*n* = 81) and *dfrA14* (*n* = 65). The remaining isolates carried eight additional variants of *dfrA*. Only one isolate harbored a combination of two different genes (*dfrA1;dfrA12*). The single ME associated with prediction of trimethoprim resistance was due to the presence of *dfrA14* without phenotypic consequences.

### Resistance to Phenicols

Genes linked to chloramphenicol resistance were identified in 215 isolates (6.16%) (**Supplementary Table S2**). Efflux pump genes were found in 194 isolates: *floR* (*n* = 147) and/or *cmlA1* (*n* = 67). All four MEs were associated with the presence of *cmlA1*. Chloramphenicol acetyltransferase genes of the *catA*- or *catB*-type were detected in 32 isolates. Eleven isolates harbored genes encoding both an efflux pump and an acetyltransferase.

### Multidrug Resistance

Out of a total 3,491 isolates, 1,301 (37.27%) were phenotypically resistant to at least one antimicrobial of the testing panel (**Table 3**). For the two most common serovars, *S*. Typhimurium and *S*. Enteritidis, this applied to 568/827 (68.68%) and 130/294 (44.22%) isolates, respectively, and for *S.* Kentucky to 82/109 isolates (75.23%).

**Table 3:** Most common combinations of antimicrobial resistance phenotypes and genotypes in non-typhoidal *Salmonella enterica* for all serovars, *S.* Typhimurium and *S.* Enteritidis.

Serovar	Antimicrobial classes	Number of isolates (%)	Most common phenotypic combination (number of isolates)	Most common genotypic combination (number of isolates)
Total (*n* = 3491)	0	2190 (62.73)	–	–
	1/2	453 (12.98)	<CIP (196)	*gyrA*[87:D-Y] (52)
	3/4	514 (14.72)	AMP/STR/SUL/TET (231)	*sul2*;*strA-strB*;*tet*(A);*bla*_TEM-1_ (219)
	5/6/7	315 (9.02)	AMP/CHL/STR/SUL/TET (37)	*sul1*;*tet*(G);*aadA17*;*aadA2*;*floR*;*bla*_PSE-1_/*bla*_CARB-2_(30)
	8/9	19 (0.54)	AMP/CAZ/CHL/< CIP/CPR/CTX/ETP/FOX/GEN/STR/SUL/TET/ TMP/TOB (1)	*qnrS1;sul1;sul2;sul3;strA-strB;tet*(M)*;tet*(A)*;dfrA12;aac(3)-Iva;aadA2;aadA12;aph(4)-Ia;cml1;floR;bla*_CMY -2_*;bla*_TEM-1_ (1)
*S*. Typhimurium (*n* = 827)	0	259 (31.32)	–	–
	1/2	101 (12.21)	TET (40)	*tet*(A) (38)
	3/4	317 (38.33)	AMP/STR/SUL/TET (230)	*sul2*;*strA-strB*;*tet*(A);*bla*_TEM-1_ (220)
	5/6/7	138 (16.69)	AMP/CHL/STR/SUL/TET (33)	*sul1*;*tet*(G);*aadA17*;*aadA2*;*floR*;*bla*_PSE-1_/*bla*_CARB-2_(28)
	8/9	12 (1.45)	AMP/CAZ/CHL/< CIP/CPR/CTX/ETP/FOX/GEN/STR/SUL/TET/ TMP/TOB (1)	*qnrS1;sul1;sul2;sul3;strA-strB;tet*(M)*;tet*(A)*;dfrA12;aac(3)-Iva;aadA2;aadA12;aph(4)-Ia;cml1;floR;bla*_CMY -2_*;bla*_TEM-1_ (1)
*S*. Enteritidis (*n* = 294)	0	164 (55.78)	–	–
	1/2	117 (39.80)	<CIP (87)	*gyrA*[87:D-Y] (24)
	≥3	13(4.42)	AMP/ < CIP/STR/SUL/TET (4)	*gyrA*[87:D-N];*sul2*;*strA-strB*;*tet*(A);*bla*_TEM-1_ (3)
*S*. Kentucky (*n* = 109)	0	27 (24.77)	–	*-*
	1/2	12 (11.01)	AMP/> CIP (5)	*gyrA*[83:S-F;87:D-Y]*;parC*[57:T-S;80:S-I]*;bla*_TEM-1_ (4)
	3/4	19 (17.43)	AMP/> CIP/SUL/TET (11)	*gyrA*[83:S-F;87:D-Y]*;parC*[57:T-S;80:S-I]*;sul1;tet*(A)*;aadA7;bla*_TEM-1_ (3)
	5/6/7	51 (46.79)	AMP/> CIP/GEN /STR/SUL/TET (24)	*gyrA*[83:S-F;87:D-Y]*;parC*[57:T-S;80:S-I]*;sul1;tet*(A)*;aac(3)-Id;aadA7;bla*_TEM-1_ (20)

*AMP, ampicillin; FOX, cefoxitin; CTX, cefotaxime; CAZ, ceftazidime; CPR, cefpirome; ETP, ertapenem; CHL, chloramphenicol; GEN, gentamicin; STR, streptomycin; TOB, tobramycin; SUL, sulphonamides; TET, tetracycline; TMP, trimethoprim; <CIP, ciprofloxacin MIC 0.06–0.25 mg/L; >CIP, ciprofloxacin MIC > 0.5 mg/L. Values in the column antimicrobial class denote the numbers of antimicrobial classes the isolates are resistant to.*

MDR, i.e., resistance to three or more antimicrobial classes, was observed in 848 of all the NTS isolates (24.29%), 467 *S*. Typhimurium (56.47%), 70 *S*. Kentucky (64.22%) and only 13 *S*. Enteritidis (4.42%). One *S*. Typhimurium isolate exhibited resistance to all nine antimicrobial classes tested.

Detected in 231 isolates overall (6.62%), resistance to ampicillin, streptomycin, sulphonamides and tetracyclines was the most commonly occurring MDR profile, all but one isolate were *S*. Typhimurium. In 219 isolates with this profile, the underlying genotype was *bla*_TEM-1,_ *strA-strB sul2, tet*(A). For *S*. Enteritidis, decreased susceptibility to ciprofloxacin was observed most frequently (*n* = 87) with *gyrA*[87:D-Y] being the most common genotypic determinant (*n* = 24). The majority of resistant *S*. Kentucky showed phenotypic resistance to ampicillin, ciprofloxacin, gentamicin, streptomycin, sulphonamides and tetracyclines (*n* = 24). In 20 isolates, this profile was based on the presence of *bla*_TEM-1,_ *gyrA[83:S-F;87:D-Y], parC[57:T-S;80:S-I], aac(3)-Id, aadA7, sul1* and *tet*(A) (*n* = 20). Thirty-three *S*. Typhimurium isolates (3.99%) exhibited the penta-resistant phenotype with resistance to ampicillin, chloramphenicol, streptomycin, sulphonamides and tetracyclines. Of these, 28 carried a combination of *bla_PSE-1_/bla*_CARB-2,_ *floR, aadA17, aadA2, sul1* and *tet*(G).

### AMR and International Travel

**FIGURE 1:**
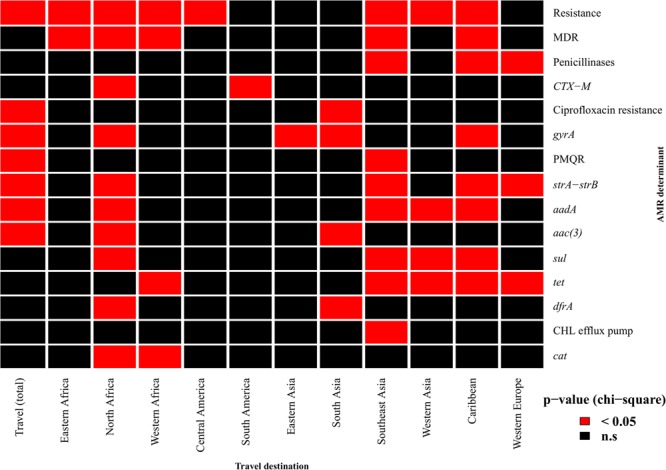
Association of resistance determinants and travel history. Red cells indicate a significant association (*p*-value < 0.05) between patient travel and the occurrence of phenotypic resistance to at least one antimicrobial, multidrug resistance (MDR) or the presence of specific resistance determinants. *gyrA* denotes isolates with single mutations in the gene responsible for reduced ciprofloxacin susceptibility. Only travel destinations for which there was an association with at least one resistance determinant are shown. PMQR, plasmid-mediated quinolone resistance; CHL, chloramphenicol.

Travel history data was available for 1,070 isolates (30.65%) (**Supplementary Table S3**). The proportion of isolates resistant to at least one antimicrobial of the testing panel was significantly higher for isolates known to be travel-associated (*p* = 4.5 × 10^-5^) (**Figure 1**). MDR, on the other hand, was correlated with travel to specific regions, namely Eastern Africa (*p* = 0.04), North Africa (*p* = 0.005), Western Africa (*p* = 0.03), Southeast Asia (*p* = 3.4 × 10^-5^) and the Caribbean (*p* = 1.3 × 10^-4^). ESBL genes were more likely to be found in isolates related to travel to North Africa (*p* = 0.01) and South America (*p* = 0.03). Mutations and acquired genes conferring decreased susceptibility or resistance to ciprofloxacin were more likely to occur in travel-associated isolates (resistance-conferring mutations: *p* = 8.5 × 10^-6^; single *gyrA* mutations: *p* = 4.5 × 10^-9^; PMQRs: *p* = 1.3 × 10^-7^). The presence of genes conferring ciprofloxacin resistance was associated with travel to Southern Asia (*p* = 5.6 × 10^-6^). Determinants of aminoglycoside resistance were more prevalent in travel-related isolates, particularly for travel destinations in North Africa, Asia and the Caribbean. The presence of sulphonamide and tetracycline resistance genes was linked to travel to Southeast and Western Asia and the Caribbean while *dfrA* genes were commonly found in isolates associated with travel to North Africa (*p* = 5.2 × 10^-7^) and South Asia (*p* = 0.01). Furthermore, travel to North Africa or Southeast Asia was a risk factor for acquisition of isolates carrying chloramphenicol resistance genes (*p* = 0.009 and *p* = 4.7 × 10^-10^, respectively).

## Discussion

The implementation of WGS for surveillance of enteric pathogens has revolutionized the work of public health laboratories, as it allows inference of a multitude of pathogen characteristics in a single sequencing run, which would traditionally require a series of independent laboratory tests. A prominent example of the added value provided by WGS is the generation of AMR profiles from the sequences in real-time.

WGS has previously proven successful for prediction of AMR profiles in a variety of gastrointestinal pathogens, including *Shigella sonnei* (Sadouki et al., 2017), *Escherichia coli* (Stoesser et al., 2013; Tyson et al., 2015; Day et al., 2017a), *S*. Typhi (Day et al., 2017b) and smaller datasets of NTS (Zankari et al., 2013; Nair et al., 2016; McDermott et al., 2016). Our present comparison of phenotypic susceptibility testing and genotypic prediction of AMR profiles based on WGS data for a much larger dataset, comprising 3,491 NTS isolates, identified 88 discordant results (0.17%) out of a possible 52,365 isolate/antimicrobial combinations, with the AMR profiles of 3,415 isolates (97.82%) completely matching for both approaches. Zankari et al. (2013) observed complete agreement of the two approaches for fifty *S*. Typhimurium isolates but only when excluding ciprofloxacin from the testing panel. Similar to our results, McDermott et al. (2016) found lower sensitivity and specificity for prediction of streptomycin resistance than for other antimicrobials tested.

Despite being an invaluable tool for surveillance purposes, AMR prediction based on WGS data is not yet deemed suitable to guide treatment choices (Ellington et al., 2017). Many MEs, where an isolate is phenotypically susceptible but carries genetic resistance determinants, seem to be associated with the breakpoints used for phenotypic testing. In some cases, the MICs are just below the recommended breakpoints but slight technical variations of the agar dilution method are possible so that the isolate would be falsely classified as susceptible. This seems to be an issue especially when testing for streptomycin resistance (Garcia-Migura et al., 2012), which would explain the relatively large number of mismatches in the present study. Recently, it has been suggested to adapt the breakpoint values to take into account MICs associated with the presence of specific resistance determinants (Tyson et al., 2017). Additionally, many of the resistance genes detected by the algorithm are plasmid-encoded but phenotypic susceptibility testing was carried out retrospectively. During storage and sub-culture of the isolates plasmids may be lost. Thus, genes detected during sequencing after initial cultivation might not be present when retrospective phenotypic testing is performed on a different colony. Furthermore, silent resistance genes, such as *bla*_CMY -2_ and *tet* variants, have been observed previously in *Salmonella* (Heider et al., 2009; Adesiji et al., 2014). Other genes, such as the *aac(6′)* variants, are normally silent and only become transcriptionally active in rare cases (Magnet et al., 1999).

The other mismatch category, the VMEs, where an isolate is genotypically predicted to be susceptible but exhibits phenotypic resistance, highlight the importance of active curation of the resistance gene database used for genotypic prediction. Mismatches are likely based on the presence of resistance determinants not included in the reference database used for prediction or on novel, unknown resistance mechanisms, the genetic determinants of which have not yet been described. Our pipeline, for instance, does not detect impermeability or efflux pump genes potentially contributing to ciprofloxacin resistance (Hopkins et al., 2005). Continuous scanning for new research findings should be carried out to enable identification of novel resistance mechanisms. These novel mechanisms will then be incorporated into the reference databases to maintain a high level of prediction sensitivity. Only recently, for example, computational methods identified previously unknown *qnr*-type fluoroquinolone resistance genes (Boulund et al., 2017). Despite these issues, the overall ME and VME rates of 0.13 and 0.04%, respectively, obtained in this study fall below the cut-offs of 3 and 1.5% from the US Food and Drug Administration for authorizing new susceptibility testing devices (FDA, 2009).

Specificity and sensitivity of ciprofloxacin resistance prediction exceeded 99% but we only considered isolates with an MIC > 0.5 mg/L for this evaluation. Traditionally, *gyrA* mutations in combination with *parC* mutations were thought to be required for ciprofloxacin resistance (Ruiz et al., 1997) and PMQRs on their own were not considered sufficient. Indeed, in our study, the majority of isolates showing resistance carried at least two mutations in both *gyrA* and *parC*. Thirty-seven had a PMQR gene, alone or in conjunction with a single *gyrA* mutation, which would normally be expected to result in reduced susceptibility instead of full resistance. Ciprofloxacin MICs for isolates carrying PMQR genes alone were found to range between 0.25 and 1 mg/L (Garcia-Fernandez et al., 2009) so that some isolates with this profile would be classed as resistant and some as having reduced susceptibility during phenotypic testing. Although the QRDR of *gyrA* is located between amino acids 67 and 106, mutations at positions 83 and 87 are most common (Yoshida et al., 1990). In our study, none of the isolates with mutations at other positions of the QRDR alone exhibited reduced ciprofloxacin susceptibility.

It has been suggested previously that an increased use of alternative antimicrobials, such as ciprofloxacin and extended-spectrum β-lactams, favored the re-emergence of susceptibility to classical first-line drugs (Sood et al., 1999; Rahman et al., 2002). The limitation of our study was that it was biased toward serovars selected for their known high resistance rates, and therefore not a true representation of the expected serovar distribution in England and Wales over this time frame. We were therefore unable to assess changes in incidence of resistance to specific antimicrobials over the years. However, moving forward, genome-derived AMR profiling will provide a robust framework to explore longitudinal trends.

A worrying trend is the increase in resistance to extended-spectrum cephalosporins (Su et al., 2005; Mataseje et al., 2009). Since these antimicrobials are used as an alternative for treatment of invasive disease in case of resistance to ciprofloxacin, the emergence of co-resistance to both antimicrobial classes is of great concern. Co-resistance is especially prevalent in Asia (Lee et al., 2009) and was identified in thirteen isolates (0.37%) in this study, a slight increase from the 0.25% prevalence observed in the UK between 2010 and 2012 (Burke et al., 2014). Seven of the eighteen isolates carrying CTX-M-type ESBLs were associated with travel to Asia and a further seven with travel to North Africa. Similarly, as observed previously (Hopkins et al., 2007), PMQR genes were more likely to be found in isolates from patients who had traveled to Asia. Extensively drug-resistant *S*. Typhimurium, like the one isolate in this study resistant to all eleven antimicrobial classes tested, have been found in Southeast Asia before (Benacer et al., 2010; Vo et al., 2010). Unfortunately, no travel history data was available for this isolate.

In addition to providing information on AMR for the entire NTS population, WGS-based prediction was able to highlight some interesting genotypic differences between the most common serovars *S*. Typhimurium and *S*. Enteritidis and the extensively drug-resistant *S*. Kentucky: ESBL genes and *aac(3)* variants, while found in *S*. Typhimurium and *S*. Kentucky, were absent in *S*. Enteritidis. Only *S*. Kentucky carried multiple mutations in the QRDRs of *gyrA* and *parC* but PMQR genes were less common than in other serovars. A more detailed investigation of these differences might lead to a better understanding of the varying outcomes associated with infections caused by different serovars of NTS (Jones et al., 2008).

## Conclusion

This large-scale study supports the suitability of WGS-based prediction to reliably replace phenotypic susceptibility testing for rapid monitoring of emerging trends in AMR patterns in NTS and for studying the spread of AMR genes in this pathogen population. Since sequencing is routinely used in public health laboratories already, it constitutes a time-saving alternative to traditional approaches that can further our understanding of resistance mechanisms as long as constant curation of the resistance gene database used is warranted. Prediction for further antimicrobials such as macrolides, fosfomycin and colistin will be validated in the near future to increase the robustness of the pipeline. Information derived from WGS-based studies can then be used to inform public health interventions aimed at limiting further dissemination of AMR genes and thus aid in the fight against the global AMR threat.

## Author Contributions

SNa, EP, TD, PA, and GG conceived the study. SNe, MRD, MD, CJ, PA, KM, KH, NW, and TD contributed to the data analysis. SNe, CJ, and TD wrote the manuscript. All authors contributed to, read, and approved the final manuscript.

## Conflict of Interest Statement

The authors declare that the research was conducted in the absence of any commercial or financial relationships that could be construed as a potential conflict of interest.

## Footnotes

1 www.lahey.org

2 https://www.ncbi.nlm.nih.gov/pathogens/beta-lactamase-data-resources

3 http://arpcard.mcmaster.ca

4 https://cge.cbs.dtu.dk/services/data.php

5 http://www.eucast.org/fileadmin/src/media/PDFs/EUCAST_files/Breakpoint_tables/v_8.0_Breakpoint_Tables.pdf

## Supplementary Material

TABLE S1 The genotypic resistance profile derived for each isolate in the study and the associated SRA accession of the deposited genome.

TABLE S2 Resistance genes detected in non-typhoidal *Salmonella enterica*, their prevalence in all serovars, *S.* Typhimurium, *S.* Enteritidis, and *S.* Kentucky and the antimicrobial class to which they confer resistance.

TABLE S3 Association of resistance determinants and travel history. Values in cells denote the number of isolates for which the travel history of the patient coincided with the presence of phenotypic resistance, multidrug resistance (MDR) or the presence of specific resistance determinants. gyrA denotes isolates with single mutations in the gene responsible for reduced ciprofloxacin susceptibility. Only travel destinations for which there was an association with at least one resistance determinant are shown. >CIP, ciprofloxacin resistance (MIC >0.5mg/L); PMQR, plasmid-mediated quinolone resistance; CHL, chloramphenicol.
